# The G-Protein-Coupled Chemoattractant Receptor Fpr2 Exacerbates High Glucose-Mediated Proinflammatory Responses of Müller Glial Cells

**DOI:** 10.3389/fimmu.2017.01852

**Published:** 2017-12-19

**Authors:** Ying Yu, Zhiyao Bao, Xiaofei Wang, Wanghua Gong, Hui Chen, Huaijin Guan, Yingying Le, Shaobo Su, Keqiang Chen, Ji Ming Wang

**Affiliations:** ^1^Eye Institute, Affiliated Hospital of Nantong University, Nantong, China; ^2^State Key Laboratory of Ophthalmology, Zhongshan Ophthalmic Center, Sun Yat-sen University, Guangzhou, China; ^3^Cancer and Inflammation Program, Center for Cancer Research, National Cancer Institute at Frederick, Frederick, MD, United States; ^4^Department of Pulmonary & Critical Care Medicine, Ruijin Hospital, Shanghai Jiaotong University School of Medicine, Shanghai, China; ^5^Basic Research Program, Leidos Biomedical Research, Inc., Frederick, MD, United States; ^6^Institute for Nutritional Sciences, National Academy of Sciences, Shanghai, China

**Keywords:** Fpr2, fibroblast growth factor receptor 1, proliferative diabetic retinopathy, Müller glial cells

## Abstract

In proliferative diabetic retinopathy (PDR), activated Müller glial cells (MGCs) exhibit increased motility and a fibroblast-like proliferation phenotype that contribute to the formation of fibrovascular membrane. In this study, we investigated the capacity of high glucose (HG) to regulate the expression of cell surface receptors that may participate in the proinflammatory responses of MGCs. We found that MGCs express a G-protein coupled chemoattractant receptor formyl peptide receptor 2 (Fpr2) and fibroblast growth factor receptor 1 (FGFR1), which mediated MGC migration and proliferation in response to corresponding ligands. HG upregulated Fpr2 through an NF-κB pathway in MGCs, increased the activation of MAPKs coupled to Fpr2 and FGFR1, which also further enhanced the production of vascular endothelial growth factor by MGCs in the presence of HG. *In vivo*, Fpr2 was more highly expressed by retina MGCs of diabetic mice and the human counterpart FPR2 was detected in the retina MGCs in fibrovascular membrane of PDR patients. To support the potential pathological relevance of Fpr2, an endogenous Fpr2 agonist cathelin-related antimicrobial peptide was detected in mouse MGCs and the retina, which was upregulated by HG. These results suggest that Fpr2, together with FGFR1, may actively participate in the pathogenesis of PDR thus may be considered as one of the potential therapeutic targets.

## Introduction

Diabetic retinopathy (DR) is a severe complication of diabetes and the leading cause of blindness. Many abnormalities seen in the retina of diabetes patients are associated with inflammation (1). Consequently, anti-inflammatory therapies significantly reduce the progression of DR in animal models. DR is staged based on levels of severity to mild, moderate, and severe nonproliferative DR, followed by advanced proliferative DR (PDR). In PDR, proliferative neovasculature and fibrovascular tissues extend from the retina into the vitreous. Tractional forces originating within these tissues cause vitreous hemorrhage, retinal scars, and tractional retinal detachment, leading to irreversible vision loss. Although the origin and the process of fibrovascular membrane formation remain unclear, it is believed that inflammation linked to hyperglycemia is the basis for DR (2).

Diabetic epiretinal membrane cells including glial cells, particularly, Müller glial cells (MGCs) and infiltrating immune cells play a central role in the formation of DR (3). MGCs span the entire thickness of the retina and are involved in retinal glucose metabolism and regulation of blood flow (4). Under pathological conditions, MGCs act as regulators of immune and inflammatory responses (5), becoming a major source of inflammatory and angiogenetic factors (6). In addition, MGCs have the capacity to transdifferentiate into myofibroblastic cells (2). Therefore, MGCs are involved in three key steps during the progression of fibrovascular membrane formation (7): (1) translocation to above or below the retina; (2) increases in cell number through mitosis; and (3) generation of tractional forces through contraction of preexisting or newly synthesized extracellular matrices. Moreover, MGCs produce angiogenetic factors such as vascular endothelial growth factor (VEGF), which is essential for the progression of DR.

Müller glial cells sense the microenvironment changes, including those that associated with high glucose (HG) through cell surface receptors and sensors. Fibroblast growth factor (FGF) is reported to play a role in the development of DR mediated by MGCs (8–10) by promoting MGC proliferation and migration. However, the contribution of G-protein-coupled chemoattractant receptors (GPCRs) which are specialized in mediating cell chemotaxis, to MGC recruitment and adhesion in PDR remains unclear.

In this study, we report that HG not only upregulates the function of fibroblast growth factor receptor 1 (FGFR1) on mouse MGCs but also enhances the expression and function of a chemoattractant GPCR, formyl peptide receptor 2 (FPR2, Fpr2 in mice) to exacerbate MGC chemotaxis, proliferation and production of VEGF, therefore contributing to the progression of PDR.

## Materials and Methods

### Animals

To induce diabetes, 6-week-old C57BL/6 mice were given five consecutive intraperitoneal injections of streptozotocin (STZ; 60 mg/kg body wt/day) (Sigma-Aldrich). Twelve weeks after injection, mice were sacrificed and eyes were harvested and embedded in OCT for sectioning. Animal study was approved by the Animal Care and Use Committee of the National Cancer Institute at Frederick, NIH.

### Reagents

Anti-Fpr2 (GM1D6) monoclonal antibody and anti-CRAMP (anticathelin-related antimicrobial peptide) (R-170) polyclonal antibody were from Santa Cruz (Santa Cruz, CA, USA). Anti-FGFR1, antiglutamine synthetase (anti-GS) and anti-Vimentin antibodies, and an FGFR antagonist PD 173074 were from Abcam (Cambridge, UK). The Fpr2 antagonist (WRW4) was purchased from Tocris Bioscience (R&D Systems, Minneapolis, MN, USA). Mouse CRAMP (NH2-ISRLAGLLRK GGEKIGEKLKKIGQKIKNFFQ KLVPQPE-OH) was synthesized by New England Peptide LLC (Gardner, MA, USA). Mouse b-FGF was purchased from Pepro Tech (Rocky Hill, NJ, USA). Sphingosine-1-phosphate (S1P) was purchased from Cayman Chemical Company (MI, USA). Antibodies specific for total ERK1/2, ERK1/2 phosphorylated at Tyr-204, phosphor (P)-p38 MAPK, and total p38 MAPK, were purchased from Cell Signaling Technology (Beverly, MA, USA). The IκB-α inhibitor BAY 11-7082 was purchased from Selleckchem (TX, USA). fMLF was obtained from Sigma-Aldrich.

### MGC Culture

Primary mouse retinal MGCs were obtained from newborn wild-type (WT) C57BL/6J and Fpr2^−/−^ mice (11) using a modified protocol (12). Following euthanasia, mice were rapidly enucleated and their globes immersed in Dulbecco’s modified Eagle’s medium (DMEM) containing 1:1,000 penicillin/streptomycin in the dark at room temperature (RT) for 6 h. The eyeballs were subsequently rinsed in phosphate-buffered saline (PBS), incubated for 25 min at 37°C with 0.05% trypsin in 0.5 mM EDTA and 200 U/mL collagenase, and then rinsed three times with DMEM containing 10% FBS (Gibco/Invitrogen, Carlsbad, CA, USA) to terminate the digestion reaction. Retinas removed from the eyeballs and dissociated by trituration were gently pipetted out and placed in culture dishes containing DMEM/F12 (Gibco, Gaithersburg, MD, USA) with 10% FBS and 1% penicillin–streptomycin. Culture medium was replaced 24 h after seeding. At 3- to 4-day intervals, cultures were shaken vigorously to detach non-adherent cells, which were then removed from the culture by aspiration. When the remaining adherent cells reached 80% confluence, they were detached from the plastic using 0.05% trypsin, resuspended in fresh DMEM containing 10% FBS, and replanted into new flasks. Immunohistochemical staining of the adherent cells showed >98% parity MGCs evidenced by immunopositivity for GS and vimentin. Experiments with MGCs were performed with cells at passages 4–8. All cells were maintained at 37°C in 5% CO_2_, 95% air, and media were changed every 2 days. To study the effect of HG, the cells were exposed to either normal (physiological) glucose (NG) (5.5 mM) or HG (25.0 mM) concentrations for indicated time points.

### RT-PCR

An RNeasy mini kit (QIAGEN) was used to extract total mRNA from MGCs. The expression of Fpr2 and mFGFR1 was examined by RT-PCR. The primers for Fpr2 were: forward, CCT GGC CCA TGA AAA CAT AG; reverse, ACA GCA GTT GTG GCT TCC TT. The primers for mFGFR1 were: forward, GAG GGT AGA ACT GGA CAG AAA C; reverse, GAC CAA CCA ACC AAC CAA AC. β-actin primers were: forward, TGT GAT GGT GGG AAT GGG TCA GAA; reverse, TGA TGT CAC GCA CGA TTT CCC TCT. All PCR products were resolved on 1.5% agarose gel by electrophoresis and visualized after ethidium bromide staining. For quantitation, gels were scanned and the pixel intensity of each band was determined using ImageJ software (NIH Image) and normalized against the amount of β-actin.

### Chemotaxis Assays

Chemotaxis assays for MGCs were performed with 48-well chemotaxis chambers as described previously (13). Wells in the lower compartment were filled with 25–27 µl medium containing different concentration of chemoattractants. The lower compartments were then covered with 10-µm pore polycarbonate membranes (NeuroProbe, Cabin John, MD, USA), which were coated with 200 µg/ml metrigel (Corning, NY, USA). Cells in RPMI 1640 containing 1% BSA (50 µl, 1.8 × 10^6^/ml) were placed in wells of the upper compartment. After incubation of the chambers at 37°C for 180 min, the membranes were collected, removed of non-migrating cells on the upper surface of the membrane, fixed and stained with Three-Step Stain Set (Thermo). The results are expressed as the mean ± SD of migrated MGCs counted in three high powered fields of the light microcopy or as the chemotaxis index (CI), representing the fold increase in the number of migrated cells in response to chemoattractants over spontaneous cell migration (to control medium without chemoattractant). For inhibition of MGC chemotaxis by FGFR or Fpr2 antagonist, MGCs were pretreated with the Fpr2 antagonist WRW4 or the FGFR inhibitor PD 173074 for 30 min before measurement of chemotaxis.

### MGC Wound Closure

Müller glial cell wound closure was evaluated by incubating the cells in 10% FBS-containing media until confluent. Several parallel straight scratch lesions were made on the cell monolayer with a sterile 1,000 µl plastic pipette tip, producing 1 mm wide scratches. The culture media were replaced with DMEM containing 1% FBS in the absence or presence of HG. The cultures were supplemented with or without CRAMP, S1P, or b-FGF. The wound closure was determined by phase contrast microscopy at designated time points.

### Cell Proliferation

Müller glial cells (5 × 10^4^) were seeded in each well of a six-well plate and incubated for 24 h in DMEM with 10% FBS, followed by culture in the absence or presence of CRAMP or b-FGF in DMEM with 2% FBS. Cells were digested then counted after 24, 48, and 72 h under phase-contrast microscopy.

### Immunostaining

Müller glial cells were seeded at 1.0 × 10^4^ cells/well on 8-well chamber slides (Nalge Nunc, Naperville, IL, USA), washed twice with PBS and fixed for 10 min with 4% paraformaldehyde in PBS, followed by Triton X-100 (0.1%) for 15 min. The cells were then incubated with 5% BSA in PBS for 1 h followed by addition of primary antibodies (Abcam, Cambridge, UK) overnight at 4°C. Secondary antibodies coupled to Alexa Fluor^®^ 488 and Alexa Fluor^®^ 555 (Abcam) (45 min, RT) were then added to the culture. After staining with DAPI to visualize nuclei, the cells were analyzed under a fluorescence microscope (Olympus IX 71).

### Western Immunoblotting

Müller glial cells grown in 60-mm dishes to subconfluency were cultured overnight in FBS-free DMEM. After treatment with CRAMP (10^−6^ M) or b-FGF (10 ng/ml), the cells were lysed with 1× SDS sample buffer, sonicated for 15 s, and then heated at 100°C for 5 min. Cell lysates were centrifuged at 12,000 rpm (4°C) for 5 min and the protein concentration in the supernatant was measured by the Micro bicinchoninic acid protein assay system (Pierce, Rockford, IL, USA). Cell lysates were then electrophoresed on 10% SDS-PAGE-precast gels (Invitrogen, Carisbad, CA, USA) under reducing conditions and then transferred onto Immunoblot polyvinylidene membranes (Bio-Rad, Hercules, CA, USA). The membranes were blocked with 5% nonfat milk then were incubated with primary antibodies overnight at 4°C. After incubation with a HRP-conjugated secondary antibody for 1 h at RT, images were quantified using a G-BOX GeneSnap system (SYNGENE). For detection of total p38, ERK1/2 and IκB the membranes were stripped with Restore Western blot stripping buffer (Pierce, Rockford, IL, USA), followed by incubation with respective antibodies.

### Enzyme-Linked Immunosorbent Assay

Confluent MGC layer was treated with CRAMP (10^−6^ M) or b-FGF (10 ng/ml) in DMEM with 2% FBS. The conditioned media were then collected at 24 h for analysis of VEGF-A ELISA kit (eBioscience) according to the supplier’s instructions.

### Patients and Tissue Samples

Study of patient materials was conducted according to the principles of the Declaration of Helsinki and was approved by the Affiliated Hospital and the Ethical Committee of Nantong University, China. Fibrovascular membranes and vitreous from patients with PDR, macular epiretinal membrane and vitreous from patients with idiopathic macular epiretinal membrane but without diabetic ocular diseases, were collected at the Affiliated Hospital of Nantong University (Nantong, China). All patients gave informed consent before enrollment. The tissues were embedded in OCT for sectioning. Undiluted vitreous samples were collected before the start of the conventional 3 ppp vitrectomy (23 Gauge, Constellation, Alcon Instruments, Fort Worth, TX, USA) without an infusion of artificial fluid. The samples were collected by manual suction into a sterile syringe from the vitrectomy then transferred into sterile 1.5 ml Eppendorf tubes and immediately frozen at −80°C until analysis.

### Statistics

Unless otherwise specified, all experiments were performed at least three times. Data are presented as the mean ± SD. A two-tailed Student’s *t*-test or ANOVA was used for evaluating statistical significance of the difference between testing and control groups. A *P* value less than 0.05 was considered statistically significant.

## Results

### HG Upregulates the Expression of Fpr2 and FGFR1 by MGCs

**Figure 1:**
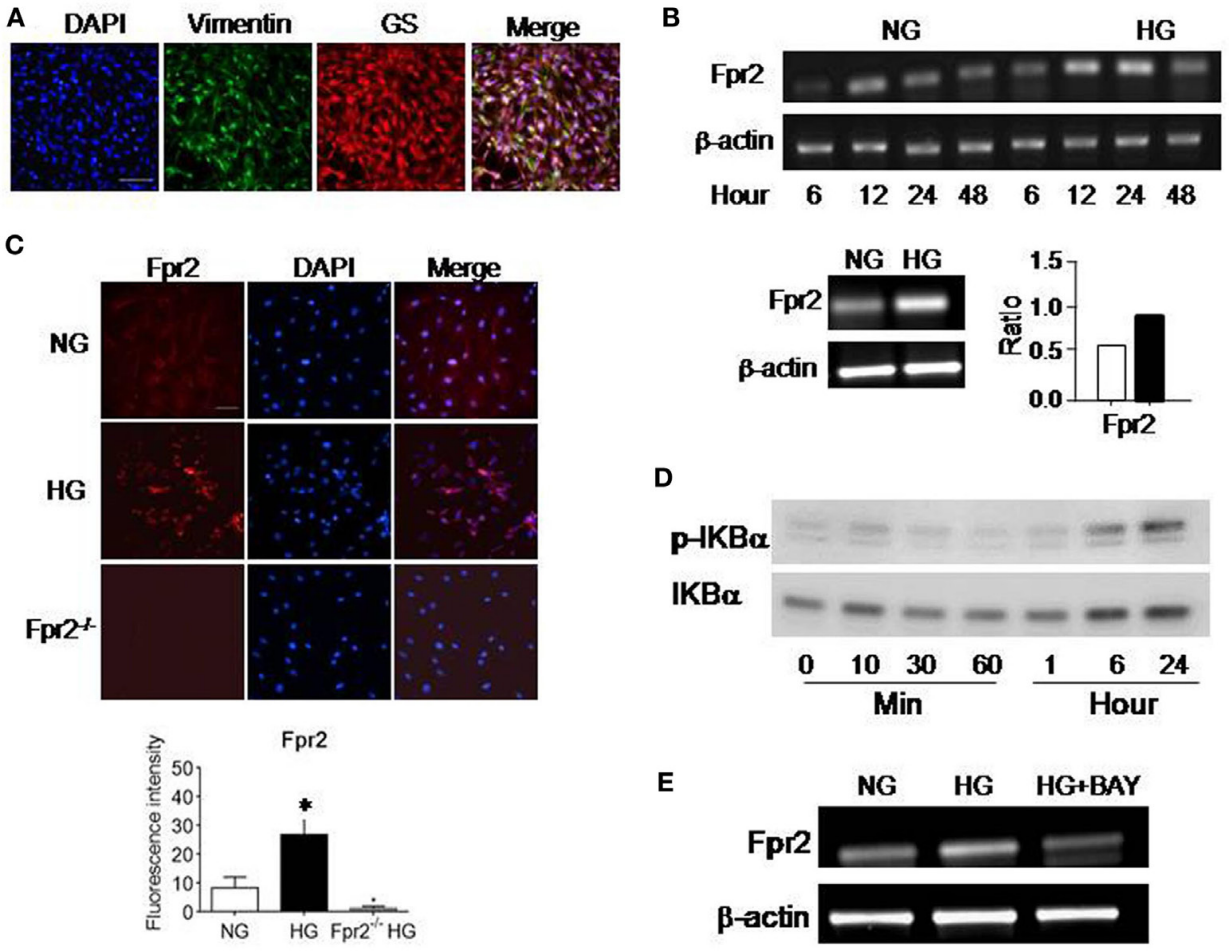
The expression of formyl peptide receptor 2 (Fpr2) by mouse Müller glial cells (MGCs). Primary mouse MGCs were exposed to normal glucose (5.5 mM, NG) or high glucose (25.0 mM, HG) for 24 h. **(A)** Staining of the cells with vimentin (green) and glutamine synthetase (GS; red) to confirm the nature of MGC. **(B)** Increased Fpr2 mRNA in HG-treated MGCs. *Indicates significantly increased Fpr2 mRNA in HG-treated MGCs compared with cells treated with NG (*p* < 0.05). **(C)** Increased level of Fpr2 shown by fluorescence intensity in HG-treated MGCs. No Fpr2 immunoreactivity was detected in MGCs from Fpr2^−/−^ mice. *Indicates significantly increased Fpr2 in fluorescence intensity in HG-treated MGCs compared with cells treated with NG (*p* < 0.05). **(D)** Western blotting showing phosphorylation of IκBα in MGCs induced by HG at the indicated time points. **(E)** The effect of IκB/NF-κB inhibitor BAY 11-7082 on Fpr2 expression by MGCs under HG for 24 h.

We first investigated the expression of Fpr2 and FGFR1 in MGCs. The nature of MGCs was confirmed by their expression of the markers vimentin and GS (Figure 1A). Fpr2 expression was analyzed with RT-PCR and immunofluorescence. As shown in Figure 1B, Fpr2 mRNA was expressed by primary mouse MGCs and significantly increased after 12 h incubation with HG. Figure 1C shows low level of Fpr2 immunoreactivity detected in MGCs cultured in NG. In contrast, after HG culture, there was a significant increase in Fpr2 immunofluorescent intensity in MGCs (Figure 1C). Fpr2 was expressed on the membrane of MGCs and the fluorescence intensity (Figures S1A,B in Supplementary Material) and the protein levels of Fpr2 on cells treated with HG were significantly higher as compared with the cells treated with NG (Figure S1C in Supplementary Material). MGCs from Fpr2^−/−^ mice did not show Fpr2 immunofluorescent staining (Figure 1C). The upregulation of Fpr2 mRNA and protein by HG was likely mediated through an NF-κB dependent signaling pathway as shown by increased IκB-α (Figure 1D) and NF-κB phosphorylation (Figures S1D and 2A in Supplementary Material) in HG-treated MGCs. The effect of HG on Fpr2 mRNA expression was attenuated by an IκB-α inhibitor BAY11-7082 (Figure 1E), which also inhibited CRAMP-induced phosphorylation of NF-κB-p65, but not p38, in MGCs (Figure S2B in Supplementary Material), indicating the specificity of BAY11-7082 on IκB/NF-κB pathway. FGFR1 expression was also analyzed with RT-PCR and immunofluorescent staining. HG did not affect FGFR1 mRNA expression, but significantly upregulated FGFR1 protein in MGCs (Figures S2C–E in Supplementary Material). Mannitol, as a control for glucose, did not show any effect of the expression and function of Fpr2 or FGFR (data not shown).

### HG Enhances the Function of Fpr2 and FGFR1 Expressed by MGCs

**Figure 2:**
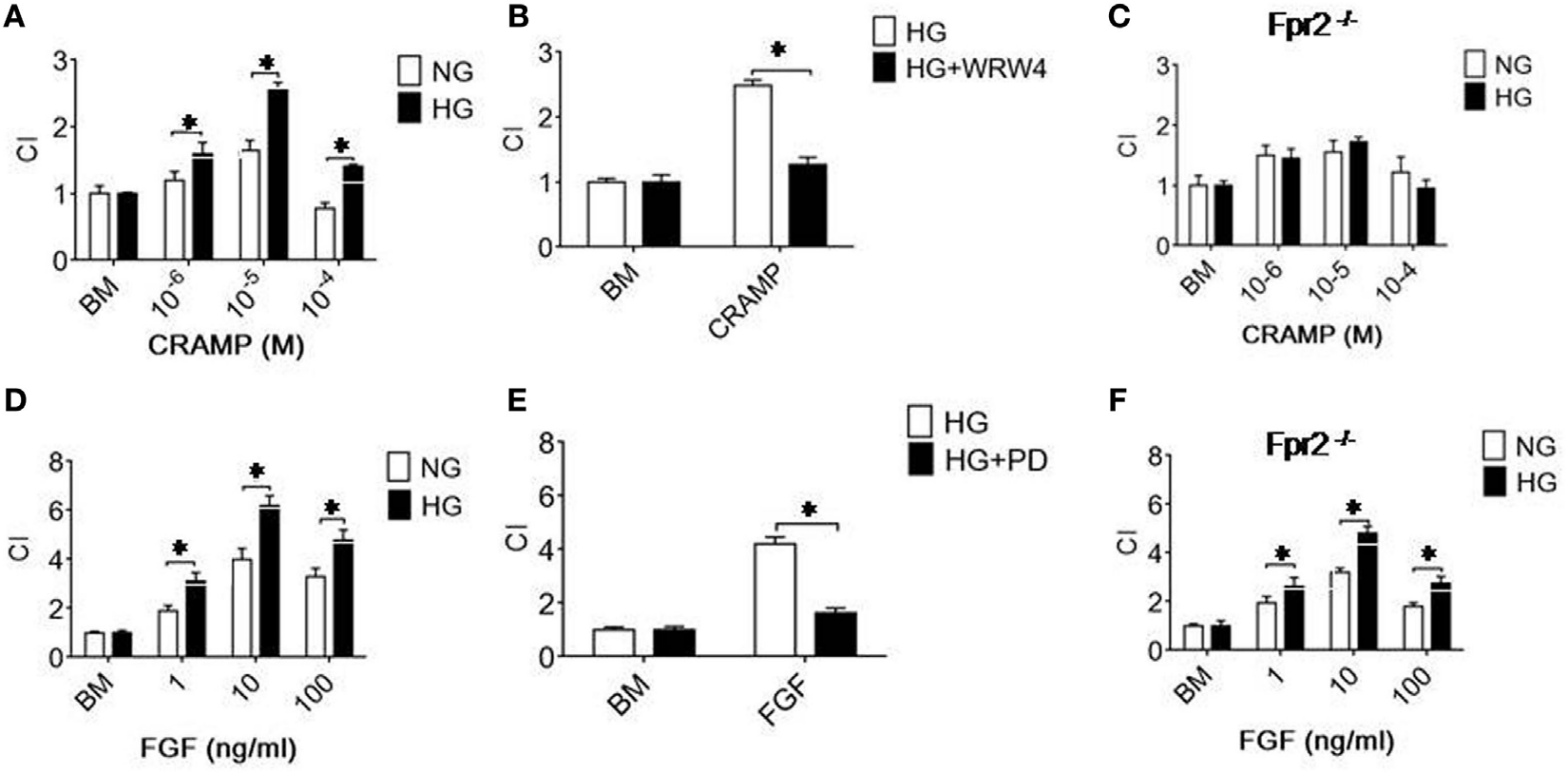
Formyl peptide receptor 2 (Fpr2)- and fibroblast growth factor receptor 1 (FGFR1)-mediated chemotaxis of Müller glial cells (MGCs). MGC chemotaxis was measured by 48-well chambers. The results are expressed by the chemotaxis index (CI) defining the fold increase in cell response to chemoattractants vs. medium control (BM). **(A)** Migration of MGCs treated with normal glucose (NG) (5.5 mM glucose) or high glucose (HG) (25.0 mM, glucose) in response to cathelin-related antimicrobial peptide (CRAMP). **(B)** Inhibition of CRAMP (10^−5^ M)-induced chemotaxis of MGCs by the Fpr2 antagonist WRW4. **(C)** Absence of chemotaxis of MGCs from Fpr2^−/−^ (RS2 KO) mice in response to CRAMP. **(D)** MGC migration in response to b-FGF. **(E)** Inhibition of b-FGF (10 ng/ml) induced chemotaxis of MGCs cultured in HG by the FGFR antagonist PD 173074 (PD). **(F)** Chemotaxis of MGCs from Fpr2^−/−^ mice in response to b-FGF. *Indicates significantly (*p* < 0.05) increased migration of MGCs cultured with HG compared with cells treated with NG. *Indicates significant (*p* < 0.05) inhibition of CRAMP-induced chemotaxis of HG-cultured MGCs.

The increased expression of Fpr2 and FGFR1 by MGCs treated with HG was associated with enhanced cell chemotaxis induced by Fpr2 and FGFR1 agonists. As shown in Figure 2A and Figure S3A in Supplementary Material, MGCs exposed to HG exhibited increased chemotaxic response to the Fpr2 ligand CRAMP as compared with cells cultured in NG. MGC chemotaxis induced by CRAMP was inhibited by the Fpr2 antagonist, WRW4 (Figure 2B; Figure S3B in Supplementary Material), indicating the involvement of Fpr2 in CRAMP-induced MGC chemotaxis. The result was also verified by using MGCs from Fpr2^−/−^ mice that failed to show any chemotaxis response to CRAMP, despite exposure to either NG or HG in culture (Figure 2C; Figure S3C in Supplementary Material). MGCs cultured in HG also showed increased chemotaxis to b-FGF, which was abolished by the FGFR inhibitor PD 173074 (Figures 2D,E; Figures S3D,E in Supplementary Material). MGCs from Fpr2^−/−^ mice retained chemotaxis response to b-FGF, indicating the receptor specificity (Figure 2F; Figure S3F in Supplementary Material) for either Fpr2 or FGFR1 expressed by MGCs.

### HG Accelerates the Rate of CRAMP- and b-FGF-Induced Wound Closure by MGCs

**Figure 3:**
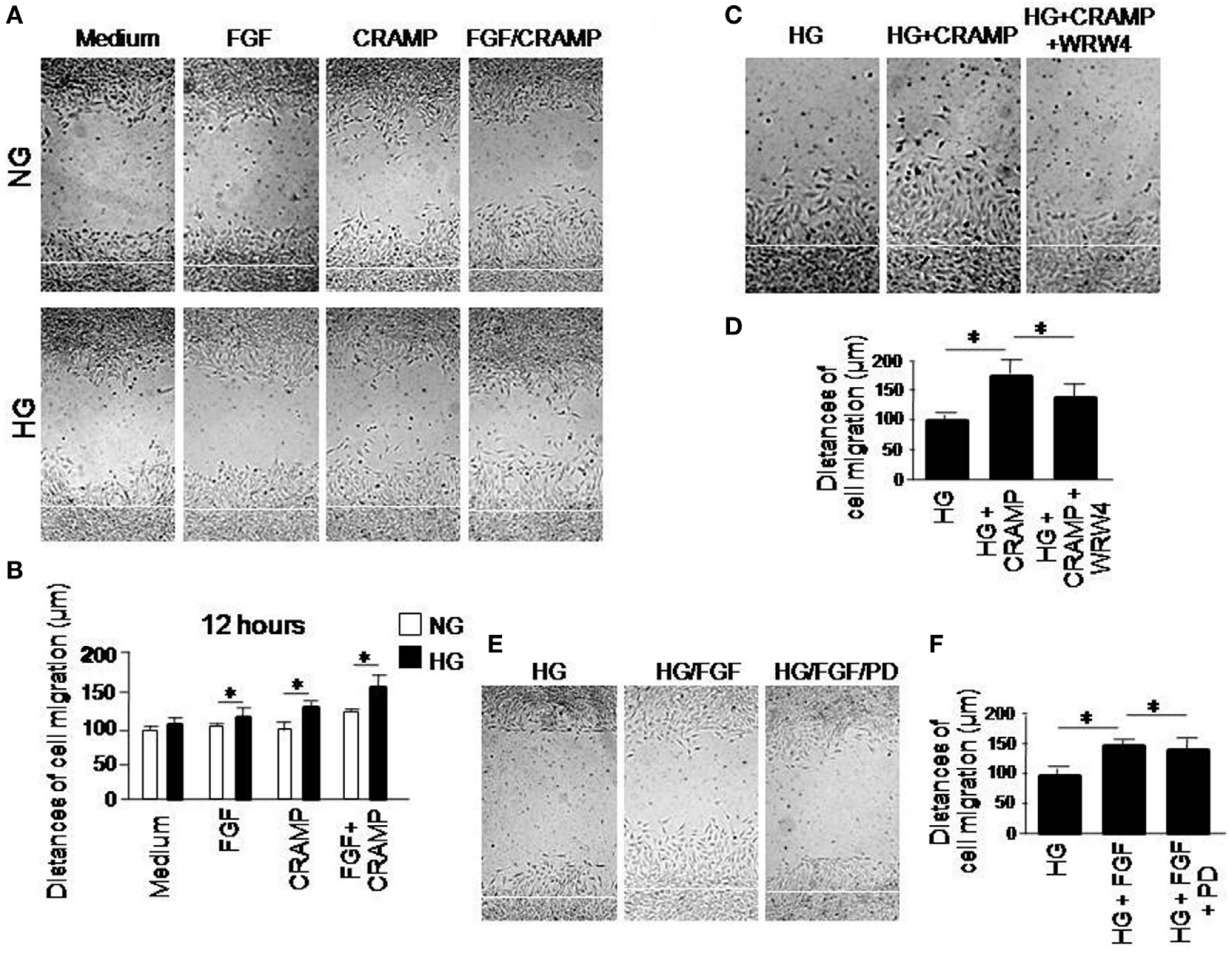
The effect of high glucose (HG) on formyl peptide receptor 2 (Fpr2)- and fibroblast growth factor receptor 1 (FGFR1)-mediated Müller glial cell (MGC) wound closure. Wound closure was measured to analyze the effect of cathelin-related antimicrobial peptide (CRAMP) (10^−6^ M) and b-FGF (10 ng/ml) on MGC migration toward the centerline of the wound under normal glucose (NG) or HG. **(A)** Wound closure measured at 12 h in the presence or absence of CRAMP or b-FGF. **(B)** Quantitative measurement of the distance of cell migration. *Indicates significantly increased rate of wound closure shown by MGCs cultured in HG compared with cells treated with NG (*p* < 0.05). **(C)** Inhibition by Fpr2 antagonist WRW4 of wound closure by MGCs under HG for 12 h. **(D)** Quantitative cell migration distance based on results shown in **(C)**. *Indicates significant (*p* < 0.05) inhibition of CRAMP-induced wound closure by MGCs cultured in HG by the WRW4. **(E)** Inhibition by FGFR1 inhibitor PD 173074 (PD) of wound closure by MGCs under HG for 12 h. **(F)** Cell distance measured based on results shown in **(E)**. *Indicates significant (*p* < 0.05) inhibition of FGF-induced wound closure by MGCs cultured in HG by the FGFR1 inhibitor PD 173074 (PD).

The motility of MGCs was further measured by a scratch-wounding model, in which HG increased the rate of wound closure by MGCs in response to CRAMP and b-FGF. Combination of CRAMP and b-FGF showed an additive effect on the capacity of MGCs to move toward the center line of the wound (Figures 3A,B). Increased MGC movement toward the wound center in the presence of HG with CRAMP or b-FGF was attenuated by WRW4 and PD 173074, the inhibitors of Fpr2 and FGFR1, respectively (Figures 3C,D) and FGFR1 (Figures 3E,F). We also compared the functions of Fpr2 ligand with S1P, which has been reported to promote the proliferation and migration of MGCs by activating sphingosine-1-phosphate receptor 1 (S1PR1) (14, 15). Our study confirmed that S1P promotes the proliferation and migration of MGCs in monolayer scratching experiments (Figures S4A,B in Supplementary Material), which were enhanced by HG (Figure S4C in Supplementary Material). However, S1P failed to induce the directional migration, i.e., the chemotaxis, of MGCs (Figures S5A,B in Supplementary Material).

### HG Enhances the Proliferation of MGCs in Response to Fpr2 Agonist CRAMP

**Figure 4:**
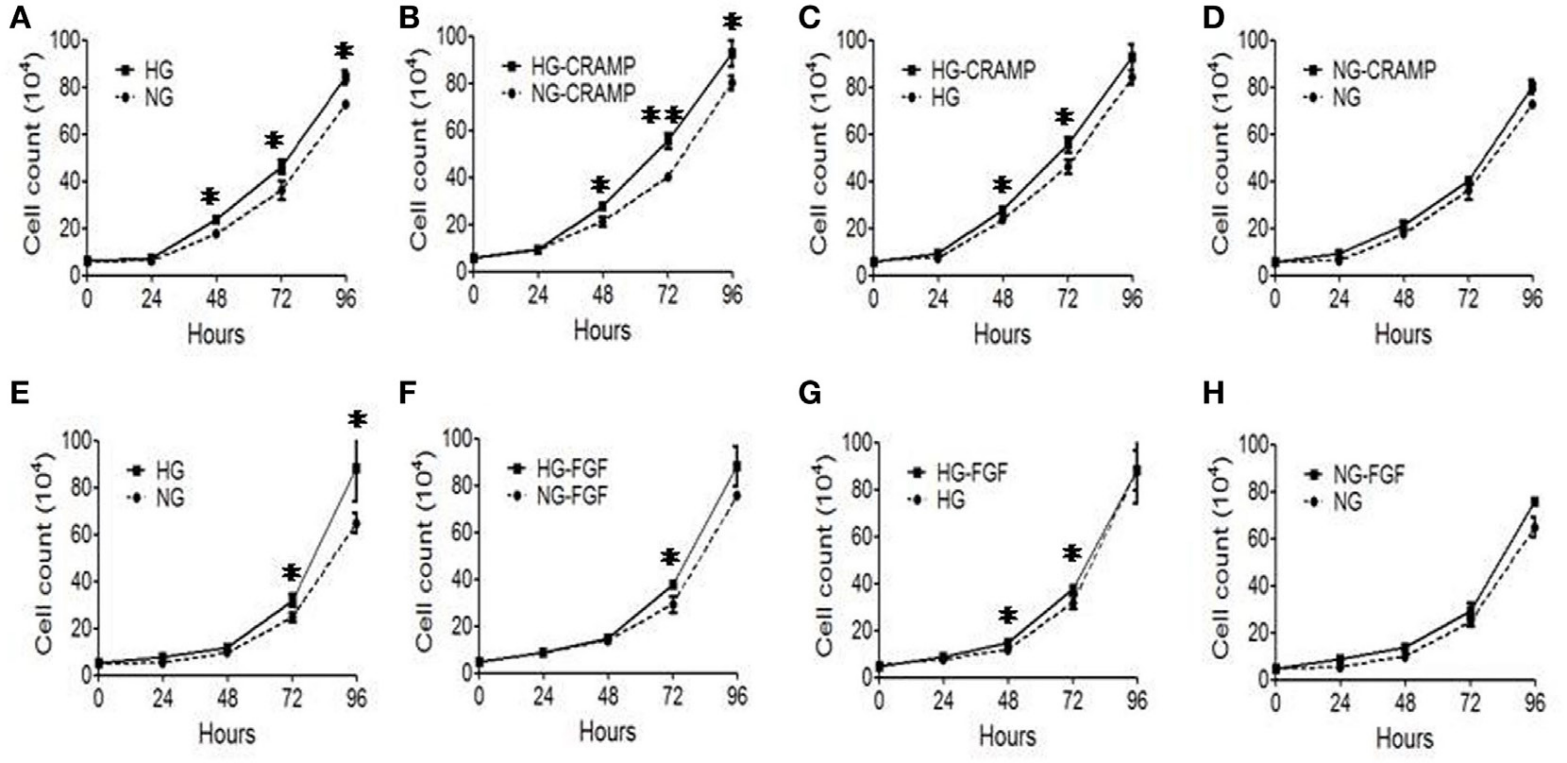
The effect of formyl peptide receptor 2 (Fpr2) and fibroblast growth factor receptor 1 (FGFR1) on Müller glial cell (MGC) proliferation in the presence or absence of high glucose (HG). MGC proliferation was examined in the presence of cathelin-related antimicrobial peptide (CRAMP) (10^−6^ M) or b-FGF (10 ng/ml) under normal glucose (NG) or HG for 24, 48, and 72 h. **(A–D)** CRAMP-induced MGC proliferation under HG or NG condition. **(E–H)** b-FGF-induced MGC proliferation under HG or NG condition. Graphs represent the mean ± SEM of triplicate samples (*n* = 3). *Indicates significantly (*p* < 0.05) increased MGC proliferation in HG compared with the cells in NG. ^#^Indicates significantly (*p* < 0.05).

We then investigated the effect of Fpr2 on MGC proliferation. MGCs cultured in the presence of HG showed a significantly increased proliferation as compared with MGCs treated with NG (Figure 4A). Treatment with CRAMP further enhanced the proliferation of MGCs cultured in HG (Figures 4B,C). However, treatment with CRAMP did not enhance the proliferation of MGCs cultured in NG (Figure 4D). b-FGF showed the similar effect on MGCs as CRAMP (Figures 4E–H). Thus, Fpr2 and FGFR1 upregulated by HG amplified proliferation of MGCs in response to their respective ligands.

### HG Increases the Activation of ERK1/2 and P38 in MGCs by Fpr2 and FGFR1 Ligands

**Figure 5:**
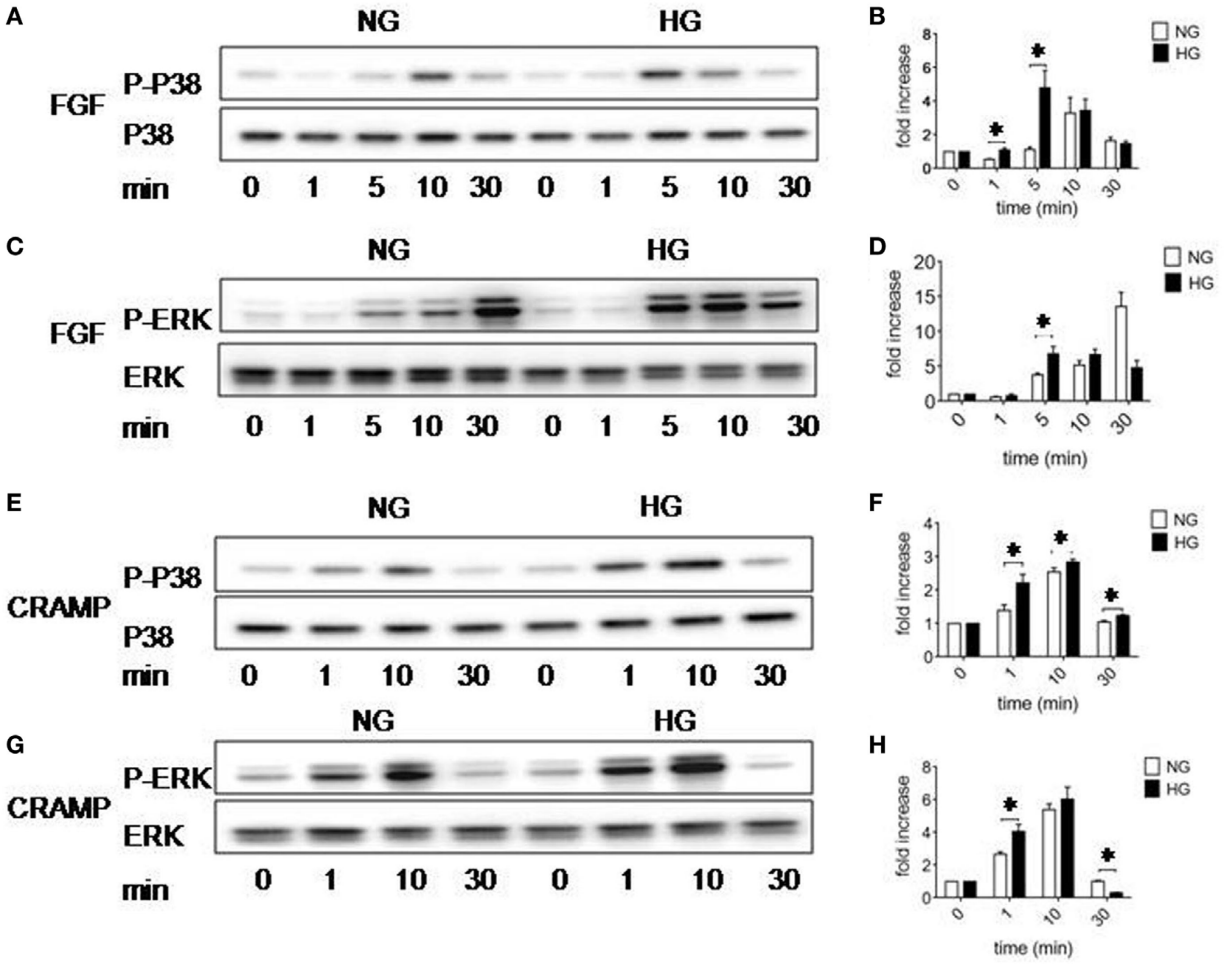
Activation of p38 and ERK1/2 MAPK in Müller glial cells (MGCs). Western blotting was performed to examine the phosphorylation of p38 and ERK1/2 MAPKs in MGCs. **(A)** p38 phosphorylation induced by fibroblast growth factor (FGF) (10 ng/ml) in MGCs cultured with normal glucose (NG) or high glucose (HG). **(B)** Densitometry quantification of phosphorylation p38 (P-p38) normalized against total p38 based on results shown in **(A)**. The results are presented as fold changes. *Indicates significantly (*p* < 0.05) increased FGF-induced P38 phosphorylation in MGCs cultured in HG compared to cells in NG. **(C)** ERK phosphorylation induced by FGF (10 ng/ml) in MGCs cultured with NG or HG. **(D)** Densitometry quantification of P-ERK normalized against total ERK. The results are presented as fold changes. *Indicates significantly (*p* < 0.05) increased ERK phosphorylation induced by FGF in MGCs cultured in HG compared to cells in NG. **(E)** p38 phosphorylation induced by cathelin-related antimicrobial peptide (CRAMP) (10^−6^ M) in MGC cultured with NG or HG. **(F)** Densitometry quantification of P-p38 normalized against total p38. The results are presented as fold changes. *Indicates significantly (*p* < 0.05) increased p38 phosphorylation induced by CRAMP in MGCs cultured in HG compared to cells in NG. **(G)** ERK phosphorylation induced by CRAMP (10^−6^ M) in MGCs cultured with NG or HG. **(H)** Densitometry quantification of P-ERK normalized against total ERK. The results are presented as fold changes. *Indicates significantly (*p* < 0.05) increased ERK phosphorylation induced by CRAMP in MGCs cultured in HG compared to cells in NG.

MAPK-signaling pathway plays an important role in multiple cellular programs such as proliferation, differentiation, chemotaxis and production of cytokines. We therefore examined the capacity of Fpr2 to activate MARKs in MGCs in the presence of HG. MGCs cultured in HG showed increased sensitivity than the cells cultured in NG by more rapid phosphorylation induced by b-FGF (5 min) (Figures 5A–D) and CRAMP (1 min) (Figures 5E–H). HG-cultured MGCs also displayed more higher levels of phosphorylation of NF-κB-p65 than NG-cultured cells in response to CRAMP (Figures S6A,B in Supplementary Material). These results suggest that HG prepares MGCs for amplified responsiveness to proinflammatory stimulants.

### MGCs Activated by HG Promotes the Production of VEGF in the Presence of Fpr2 and FCFR Agonists

**Figure 6:**
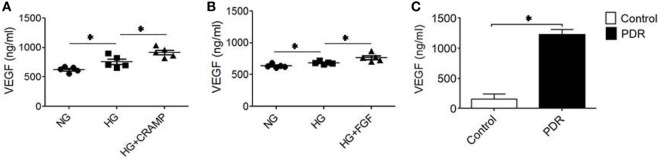
Production of vascular endothelial growth factor (VEGF) by Müller glial cells (MGCs). MGCs were treated with **(A)** cathelin-related antimicrobial peptide (CRAMP) (10^−6^ M) or **(B)** b-FGF (10 ng/ml) for 12 h followed by ELISA to measure the production of VEGF in the supernatants. *Indicates significantly increased production of VEGF (*p* < 0.05). **(C)** VEGF production in the vitreous homogenate from patients with or without diabetic retinopathy (DR). *Indicates significantly increased production of VEGF in the vitreous from patients with proliferative DR compared to patients without DR (*p* < 0.05).

Proliferative DR is characterized by rigorous neovascularization. Since MGCs are major source of VEGF critical for angiogenesis in the retina, we measured VEGF production by MGCs. HG increased the production of VEGF by MGCs, which was further enhanced by CRAMP (Figure 6A) and b-FGF (Figure 6B) treatment. Thus, MGCs activated by HG are capable of producing increased VEGF after stimulation with Fpr2 and FGFR agonists. To corroborate the clinical relevance, we measured VEGF in vitreous of patients with PDR. Figure 6C shows higher levels of VEGF in vitreous of PDR, suggesting the potential of HG to promote proinflammatory responses and angiogenesis in the retina.

### Increased Expression of Fpr2 in Diabetic Mouse Retina and in Fibrovascular Membrane from PDR Patients

**Figure 7:**
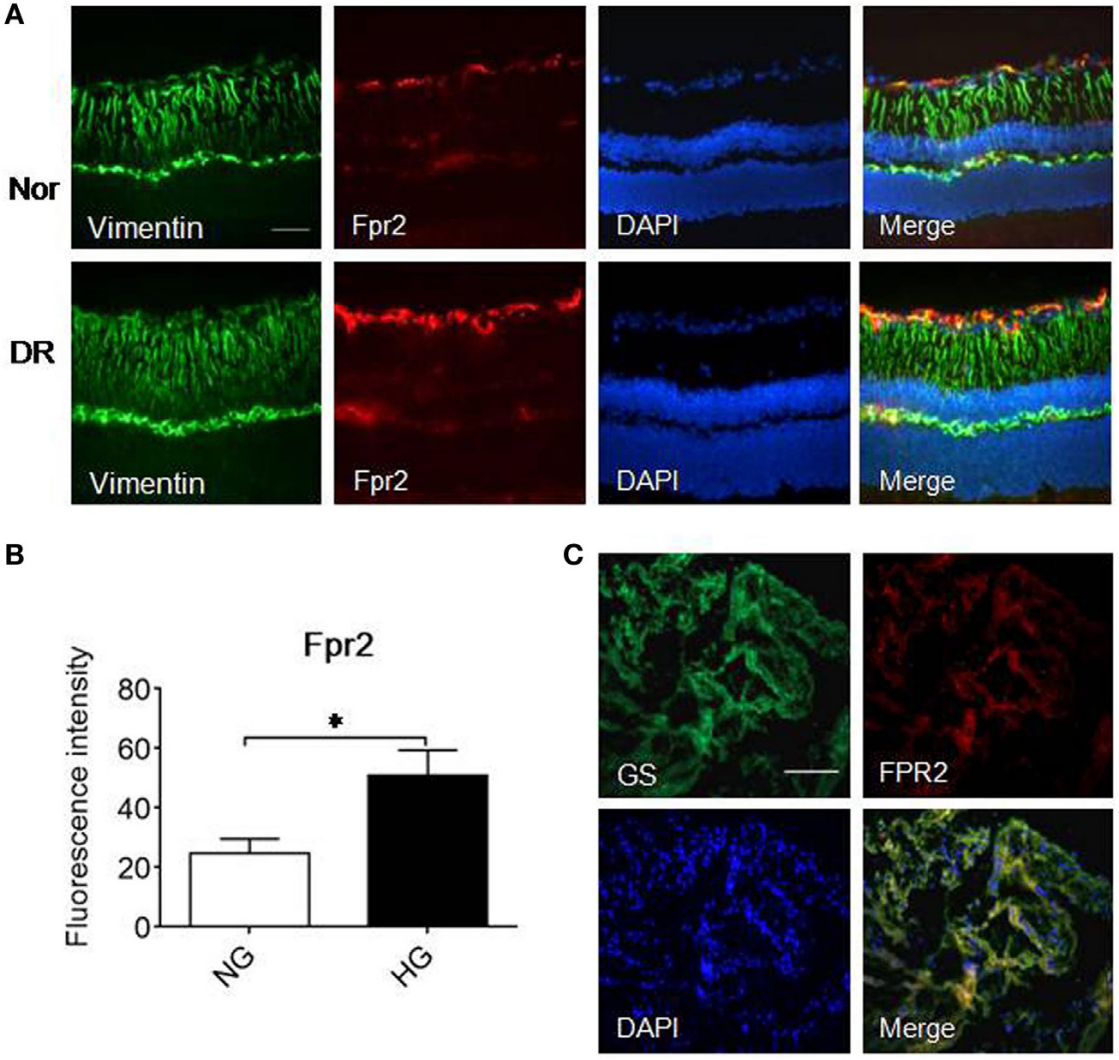
The expression of formyl peptide receptor 2 (Fpr2) in Müller glial cells (MGCs) in mice with streptozotocin (STZ)-induced diabetic retinopathy (DR) as well as FPR2 in the fibrovascular membrane in patients with proliferative DR (PDR). Immunofluorescence staining of Fpr2 (red) and vimentin (green) was performed in retinas of normal mice and mice with STZ-induced DR. FPR2 fluorescence was also examined in the fibrovascular membrane of PDR patients. **(A)** Immunofluorescence. Green: vimentin; red: Fpr2; blue: nucleus. **(B)** Fpr2 immunofluorescence intensity quantified based on images in **(A)**. *Indicates significant increased Fpr2 intensity in DR mouse retina compared with normal mouse retina (*p* < 0.05). **(C)** FPR2 immunofluorescence in the fibrovascular membrane of PDR patients, red: FPR2, green: GS. Scale bar: 50 µm.

We further examined the expression of Fpr2 in MGCs in the mouse retina. In normal mouse retina, Fpr2 was expressed in the inner nuclear layer as measured by immunofluorescence (Figure 7A). The expression of Fpr2 was markedly upregulated in the retina of diabetic mice, with increased proliferation of MGCs to form a thicker layer (Figure 7B). In patients with PDR, FPR2 was also highly expressed in the fibrovascular membrane (Figure 7C), suggesting the involvement of FPR2 in the development of fibrovascular membrane in DR.

### HG Upregulates the Expression of Fpr2 Agonist CRAMP in MGCs and in Diabetic Mouse Retina

**Figure 8:**
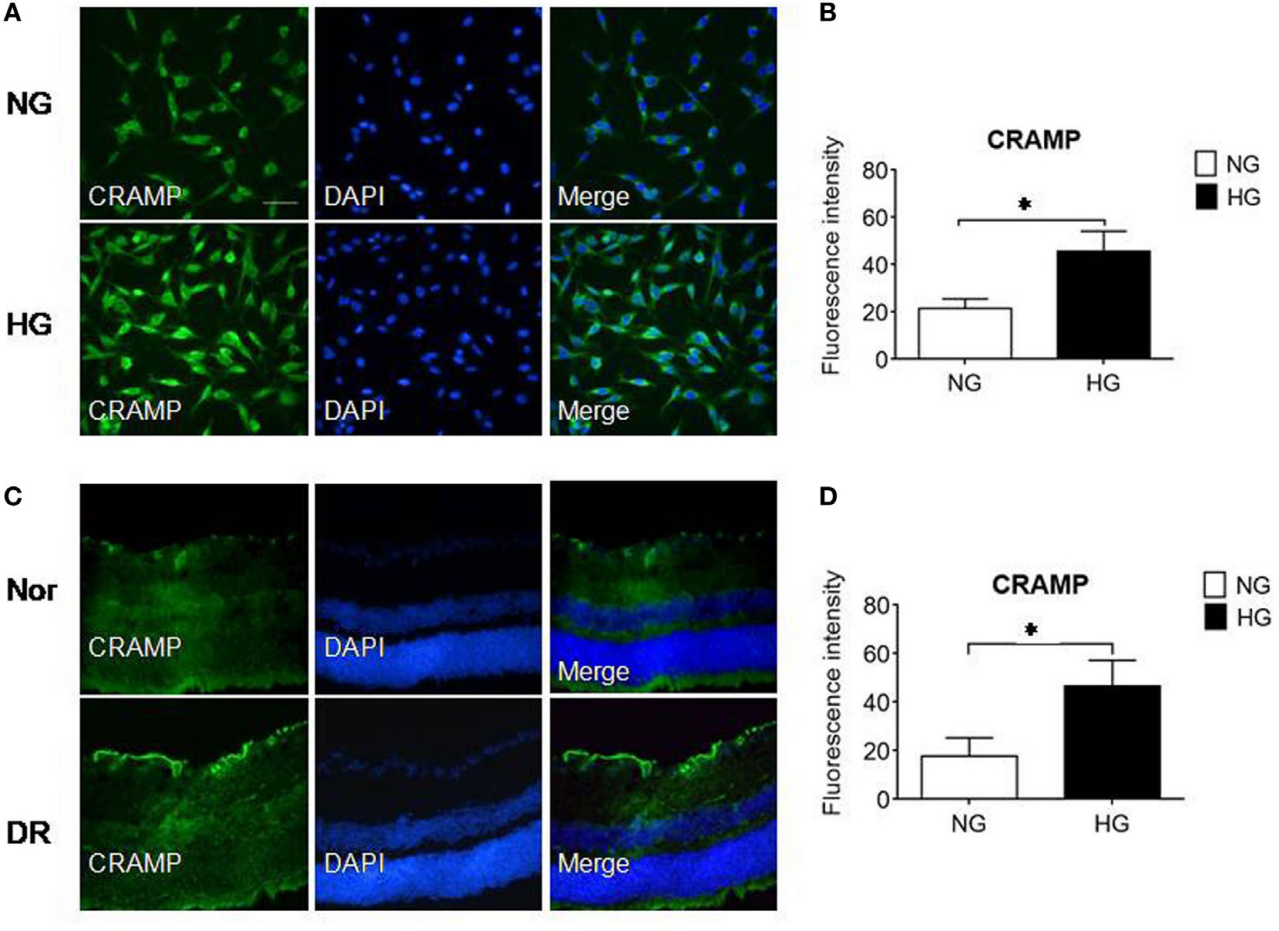
The expression of cathelin-related antimicrobial peptide (CRAMP) in Müller glial cells (MGCs) and in the retina of mice with streptozotocin (STZ)-induced diabetic retinopathy (DR). CRAMP was measured by immunofluorescence in isolated primary mouse MGCs and in the retina of normal (Nor) and diabetic (DR) mice. **(A)** Increased level of CRAMP (green) fluorescence intensity in MGCs under high glucose (HG) compared with cells under normal glucose (NG). **(B)** Relative intensity of CRAMP immunofluorescence. *Indicates significantly (*p* < 0.05) increased CRAMP in MGCs cultured in HG compared with cells in NG. **(C)** Immunofluorescence of CRAMP (green) in normal and DR mouse retinas. **(D)** Relative intensity of CRAMP immunofluorescence in mouse retinal sections. *Indicates significantly (*p* < 0.05) increased CRAMP intensity in DR mouse retina compared with normal mouse retina. Scale bar: 50 µm.

To investigate the possibility of Fpr2 expressed by MGCs to interact with endogenous agonist CRAMP in the retina, we used immunofluorescence to detect CRAMP in isolated MGCs and in the mouse retina. Figures 8A,B shows that primary MGCs under NG exposure contained a low level of CRAMP, which was significantly increased after treatment with HG. *In vivo*, CRAMP was readily detectable in normal mouse retina and its level was markedly increased in MGCs from diabetic mouse retina (Figures 8C,D). The potential participation of endogenous agonist CRAMP in the pathophysiology of retina was evidenced by the observation that MGC proliferation in the presence of HG was attenuated by the Fpr2 antagonist WRW4 in the absence of CRAMP, FGF or S1P stimulation (Figure 3C; Figures S4A,B in Supplementary Material). These observations suggest the existence of an Fpr2-CRAMP interactive axis in the retina, which may respond to HG present in diabetes to promote inflammation and angiogenesis.

## Discussion

In this study, we have shown that HG enhanced the expression and function of Fpr2 and FGFR1 in MGCs through an NF-κB dependent signaling pathway. HG promotes MGC proliferation, random migration and directional chemotaxis and VEGF secretion in response to the agonists for Fpr2 and FGFR1 that may exacerbate the pathogenesis of fibrovascular membrane formation in PDR.

Sphingosine-1-phosphate is a sphingolipid synthesized by sphingosine kinase (SphK), which has been reported to promote the proliferation and migration of MGCs by activating S1PR1, one of the five S1PR subfamily members (S1PR1-5) (14). MGCs synthesize S1P, which signals through S1P3 to activate PI3K and ERK/MAPK pathways to induce MGC migration in wound scratching assays (15). Our study confirmed that S1P promotes the proliferation and migration of MGCs in monolayer scratching experiments, which are enhanced by HG. However, while S1P increases the migration of MGCs in monolayer scratching assays where S1P is not present as a gradient, it fails to induce the directional migration, i.e., chemotaxis, of MGCs. This is distinct from the effect of Fpr2, which not only increases MGC proliferation and healing of monolayer wound under HG but also the directional migration (chemotaxis), a key event for cell recruitment under inflammatory conditions. Therefore, S1P receptors and Fpr2 may contribute to the pathogenesis of MGC-mediated DR at different disease stages.

Diabetic retinopathy differs from traditional pathogenic microbe-induced inflammation in the eye, manifesting a low-level, chronic, and atypical inflammatory process. Cytokines, chemokines, adhesion molecules, prostaglandins, and inflammatory cells including macrophages and neutrophils participate in a complex chain of events in DR (16–18). Leukocytosis is particularly present in the retinas of diabetic mice (19). In rats, leukocytosis is associated with retinal endothelial cell injury and death (20). MGCs are a major source of inflammatory mediators (21). High-throughput profiling demonstrates diabetes-induced expression of genes in MGCs that are mostly associated with inflammation (22), suggesting that MGCs contribute to inflammatory responses during exposure to HG and the development of DR. An important angiogenic factor VEGF is rapidly released from MGCs in early DR, enhancing the extension of microvessels with concomitant decrease in anti-angiogenic pigment epithelium-derived factor (23, 24). In VEGF deficient mice, diabetes-induced retinal inflammation, vascular leakage, and vascular degeneration are considerably attenuated (23). Thus, increased glucose constitutes an important initiator of proinflammatory and angiogenic cascade in diabetes-associated retinal pathology (25). HG has been shown to enhance the production of inflammatory cytokines by MGCs through the activation of p38 MAPK/NF-κB signaling pathway (21). This HG-activated pathway in MGCs also is exploited by the cells to enhance the expression and function of FPRs, which are important GPCRs to regulate innate inflammatory responses including immune cell migration in response to a variety of chemotactic agonists including CRAMP (human LL37) (26). In our study, we found that mouse MGCs express one of the FPR members Fpr2 and its expression was upregulated by exposure to HG, providing a basis for amplified inflammatory cell responses.

Formyl peptide receptors (Fprs) regulate innate inflammatory responses and mediate cell migration in response to a variety of chemotactic factors (26). Fpr2 and its human counterpart FPR2 interact with a great number of bacteria-derived and endogenous chemotactic molecular patterns. FPR2 (mouse Fpr2), which is expressed not only on myeloid cells but also colonic crypt epithelial cells, mediates N-formylpeptide–dependent epithelial cell proliferation and renewal. Colonic epithelial cells in Fpr2-deficient mice displayed delayed mucosal restoration after injury, and increased azoxymethane-induced tumorigenesis (27). By interacting with host derived agonists, Fpr2 also contributes to the healing of wounds in the skin and gastric mucosa (28, 29).

Cathelin-related antimicrobial peptide as a host-derived endogenous Fpr2 agonist exhibits direct antibacterial activity but also induces chemotaxis of myeloid cells. Recently, owing to the development of genetically engineered mouse strains, the *in vivo* role of Fpr2 and CRAMP has been increasingly recognized. Our previous study showed both Fpr2 and CRAMP control dendritic cell trafficking in inflammatory and immune responses (30, 31). The human LL-37 enhances wound healing in diabetic mice, suggesting that LL-37 actively participates in re-epithelialization and granulation of retina tissue (32). Our current study shows that both Fpr2 and CRAMP are increased in MGCs from diabetic mouse retina both *in vivo* and *in vitro*, which may attribute the Fpr2 and CRAMP axis to increased proliferation, migration, and secretion of VEGF by MGCs to aggravate DR.

b-FGF has been implicated in the development of DR mediated by MGCs. Our study found that FGFR1 is expressed by MGCs and HG further increased its functional expression. The FGFR superfamily consists of four different isoforms FGFR1–4 (33). It has been shown that *in vivo* astrocytes express FGFR1 mRNA and protein (34). In the retina, FGFR1 (SR1) was distributed mainly in the inner nuclear layer and was detected in the radial fibers of retinal MGCs. FGF2 and FGFR1 mRNA levels are greatly increased in light-induced retinal degeneration (35). MAPK-signaling through FGF receptors regulates the proliferation of MGCs and stimulates the cells to become more neuroprotective against excitotoxicity (36). Our study shows that FGFR1 similar to Fpr2 is upregulated by HG in MGCs and interestingly, Fpr2 and FGFR1 additively mediated the migration and proliferation of MGCs under HG. Thus, in PDR, it is plausible that Fpr2 and FGFR1 cooperate to mediate pro-inflammatory and angiogenic responses of MGCs and constitute potential targets for alleviating PDR.

## Ethics Statement

Study of patient materials was conducted according to the principles of the Declaration of Helsinki and was approved by the Affiliated Hospital and the Ethical Committee of Nantong University, China. Animal study was approved by the Animal Care and Use Committee of the National Cancer Institute at Frederick, NIH.

## Author Contributions

YY performed experiments, analyzed data, and drafted the manuscript; ZB performed experiments, analyzed data; KC analyzed data and edited the manuscript; WG, XW performed experiments and acquired data; YL generated Fpr2^−/−^ mice; HC, HG, and SS reviewed the manuscript; JW designed the experiments and edited and finally approved the manuscript.

## Conflict of Interest Statement

The authors declare that the research was conducted in the absence of any commercial or financial relationships that could be construed as a potential conflict of interest.

## References

[1] Tang J Kern TS. Inflammation in diabetic retinopathy. Prog Retin Eye Res (2011) 30:343–58. 10.1016/j.preteyeres.2011.05.002 21635964 PMC3433044

[2] Guidry C The role of Muller cells in fibrocontractive retinal disorders. Prog Retin Eye Res (2005) 24:75–86. 10.1016/j.preteyeres.2004.07.001 15555527

[3] Guidry C King JL Mason JO III Fibrocontractive Muller cell phenotypes in proliferative diabetic retinopathy. Invest Ophthalmol Vis Sci (2009) 50:1929–39. 10.1167/iovs.08-2475 19117921

[4] Bringmann A Pannicke T Grosche J Francke M Wiedemann P Skatchkov SN Muller cells in the healthy and diseased retina. Prog Retin Eye Res (2006) 25:397–424. 10.1016/j.preteyeres.2006.05.003 16839797

[5] Abu El-Asrar AM Mohammad G Nawaz MI Siddiquei MM Van den Eynde K Mousa A Relationship between vitreous levels of matrix metalloproteinases and vascular endothelial growth factor in proliferative diabetic retinopathy. PLoS One (2013) 8:e85857. 10.1371/journal.pone.0085857 24392031 PMC3877391

[6] Mizutani M Gerhardinger C Lorenzi M Muller cell changes in human diabetic retinopathy. Diabetes (1998) 47:445–9. 10.2337/diabetes.47.3.445 9519752

[7] Bringmann A Wiedemann P Involvement of Muller glial cells in epiretinal membrane formation. Graefes Arch Clin Exp Ophthalmol (2009) 247:865–83. 10.1007/s00417-009-1082-x 19415318

[8] Li JK Wei F Jin XH Dai YM Cui HS Li YM. Changes in vitreous VEGF, bFGF and fibrosis in proliferative diabetic retinopathy after intravitreal bevacizumab. Int J Ophthalmol (2015) 8:1202–6. 10.3980/j.issn.2222-3959.2015.06.22 26682173 PMC4651889

[9] Hueber A Wiedemann P Esser P Heimann K. Basic fibroblast growth factor mRNA, bFGF peptide and FGF receptor in epiretinal membranes of intraocular proliferative disorders (PVR and PDR). Int Ophthalmol (1996) 20:345–50. 9237137 10.1007/BF00176889

[10] Fredj-Reygrobellet D Baudouin C Negre F Caruelle JP Gastaud P Lapalus P. Acidic FGF and other growth factors in preretinal membranes from patients with diabetic retinopathy and proliferative vitreoretinopathy. Ophthalmic Res (1991) 23:154–61. 10.1159/000267115 1719460

[11] Chen K Le Y Liu Y Gong W Ying G Huang J A critical role for the g protein-coupled receptor mFPR2 in airway inflammation and immune responses. J Immunol (2010) 184:3331–5. 10.4049/jimmunol.0903022 20200280 PMC7330933

[12] Wang M Ma W Zhao L Fariss RN Wong WT Adaptive Muller cell responses to microglial activation mediate neuroprotection and coordinate inflammation in the retina. J Neuroinflammation (2011) 8:173 10.1186/1742-2094-8-173 22152278 PMC3251543

[13] Chen K Zhang L Huang J Gong W Dunlop NM Wang JM. Cooperation between NOD2 and Toll-like receptor 2 ligands in the up-regulation of mouse mFPR2, a G-protein-coupled Abeta42 peptide receptor, in microglial cells. J Leukoc Biol (2008) 83:1467–75. 10.1189/jlb.0907607 18299458

[14] Hanson MA Roth CB Jo E Griffith MT Scott FL Reinhart G Crystal structure of a lipid G protein-coupled receptor. Science (2012) 335:851–5. 10.1126/science.1215904 22344443 PMC3338336

[15] Simon MV Prado Spalm FH Politi LE Rotstein NP Sphingosine-1-Phosphate Is a Crucial Signal for Migration of Retina Muller Glial Cells. Invest Ophthalmol Vis Sci (2015) 56:5808–15. 10.1167/iovs.14-16195 26325420

[16] Ascaso FJ Huerva V Grzybowski A. The role of inflammation in the pathogenesis of macular edema secondary to retinal vascular diseases. Mediators Inflamm (2014) 2014:432685. 10.1155/2014/432685 25152567 PMC4134827

[17] Khalfaoui T Lizard G Ouertani-Meddeb A Adhesion molecules (ICAM-1 and VCAM-1) and diabetic retinopathy in type 2 diabetes. J Mol Histol (2008) 39:243–9. 10.1007/s10735-007-9159-5 18165914

[18] Grant MB Afzal A Spoerri P Pan H Shaw LC Mames RN. The role of growth factors in the pathogenesis of diabetic retinopathy. Expert Opin Investig Drugs (2004) 13:1275–93. 10.1517/13543784.13.10.1275 15461557

[19] Vincent JA Mohr S. Inhibition of caspase-1/interleukin-1beta signaling prevents degeneration of retinal capillaries in diabetes and galactosemia. Diabetes (2007) 56:224–30. 10.2337/db06-0427 17192486

[20] Joussen AM Murata T Tsujikawa A Kirchhof B Bursell SE Adamis AP. Leukocyte-mediated endothelial cell injury and death in the diabetic retina. Am J Pathol (2001) 158:147–52. 10.1016/S0002-9440(10)63952-1 11141487 PMC1850259

[21] Liu X Ye F Xiong H Hu DN Limb GA Xie T IL-1beta Induces IL-6 production in retinal Muller cells predominantly through the activation of P38 MAPK/NF-kappaB signaling pathway. Exp Cell Res (2014) 331(1):223–31. 10.1016/j.yexcr.2014.08.040 25239226

[22] Gerhardinger C Costa MB Coulombe MC Toth I Hoehn T Grosu P Expression of acute-phase response proteins in retinal Muller cells in diabetes. Invest Ophthalmol Vis Sci (2005) 46:349–57. 10.1167/iovs.04-0860 15623795

[23] Wang J Xu X Elliott MH Zhu M Le YZ Muller cell-derived VEGF is essential for diabetes-induced retinal inflammation and vascular leakage. Diabetes (2010) 59:2297–305. 10.2337/db09-1420 20530741 PMC2927953

[24] Penn JS Madan A Caldwell RB Bartoli M Caldwell RW Hartnett ME. Vascular endothelial growth factor in eye disease. Prog Retin Eye Res (2008) 27:331–71. 10.1016/j.preteyeres.2008.05.001 18653375 PMC3682685

[25] Yego EC Vincent JA Sarthy V Busik JV Mohr S Differential regulation of high glucose-induced glyceraldehyde-3-phosphate dehydrogenase nuclear accumulation in Muller cells by IL-1beta and IL-6. Invest Ophthalmol Vis Sci (2009) 50:1920–8. 10.1167/iovs.08-2082 19060282

[26] Prevete N Liotti F Marone G Melillo RM de Paulis A. Formyl peptide receptors at the interface of inflammation, angiogenesis and tumor growth. Pharmacol Res (2015) 102:184–91. 10.1016/j.phrs.2015.09.017 26466865

[27] Chen K Liu M Liu Y Yoshimura T Shen W Le Y Formylpeptide receptor-2 contributes to colonic epithelial homeostasis, inflammation, and tumorigenesis. J Clin Invest (2013) 123:1694–704. 10.1172/JCI65569 23454745 PMC3613917

[28] de Paulis A Prevete N Rossi FW Rivellese F Salerno F Delfino G Helicobacter pylori Hp(2-20) promotes migration and proliferation of gastric epithelial cells by interacting with formyl peptide receptors in vitro and accelerates gastric mucosal healing in vivo. J Immunol (2009) 183:3761–9. 10.4049/jimmunol.0900863 19692643

[29] Liu M Chen K Yoshimura T Liu Y Gong W Le Y Formylpeptide receptors mediate rapid neutrophil mobilization to accelerate wound healing. PLoS One (2014) 9:e90613. 10.1371/journal.pone.0090613 24603667 PMC3946181

[30] Chen K Liu M Liu Y Wang C Yoshimura T Gong W Signal relay by CC chemokine receptor 2 (CCR2) and formylpeptide receptor 2 (Fpr2) in the recruitment of monocyte-derived dendritic cells in allergic airway inflammation. J Biol Chem (2013) 288:16262–73. 10.1074/jbc.M113.450635 23603910 PMC3675565

[31] Chen K Xiang Y Huang J Gong W Yoshimura T Jiang Q The formylpeptide receptor 2 (Fpr2) and its endogenous ligand cathelin-related antimicrobial peptide (CRAMP) promote dendritic cell maturation. J Biol Chem (2014) 289:17553–63. 10.1074/jbc.M113.535674 24808174 PMC4067191

[32] Carretero M Escamez MJ Garcia M Duarte B Holguin A Retamosa L In vitro and in vivo wound healing-promoting activities of human cathelicidin LL-37. J Invest Dermatol (2008) 128:223–36. 10.1038/sj.jid.5701043 17805349

[33] Turner N Grose R Fibroblast growth factor signalling: from development to cancer. Nat Rev Cancer (2010) 10:116–29. 10.1038/nrc2780 20094046

[34] Reilly JF Kumari VG. Alterations in fibroblast growth factor receptor expression following brain injury. Exp Neurol (1996) 140:139–50. 10.1006/exnr.1996.0124 8690057

[35] Guillonneau X Regnier-Ricard F Laplace O Jonet L Bryckaert M Courtois Y Fibroblast growth factor (FGF) soluble receptor 1 acts as a natural inhibitor of FGF2 neurotrophic activity during retinal degeneration. Mol Biol Cell (1998) 9:2785–802. 10.1091/mbc.9.10.2785 9763444 PMC25554

[36] Fischer AJ Scott MA Ritchey ER Sherwood P Mitogen-activated protein kinase-signaling regulates the ability of Muller glia to proliferate and protect retinal neurons against excitotoxicity. Glia (2009) 57:1538–52. 10.1002/glia.20868 19306360 PMC2775435

