# Supplementary material for: The G-Protein-Coupled Chemoattractant Receptor Fpr2 Exacerbates High Glucose-Mediated Proinflammatory Responses of Müller Glial Cells
Source: Front Immunol. 2017 Dec 19;8:1852. doi: 10.3389/fimmu.2017.01852 (PMC5742138; doi:10.3389/fimmu.2017.01852)
Supplement: Supplementary file 1 [file Data_Sheet_1.doc]

Supplementary Data

**The G-protein-coupled chemoattractant receptor Fpr2 exacerbates high glucose-mediated proinflammatory responses of Müller glial cells**

Ying Yu, Zhiyao Bao, Xiaofei Wang, Wanghua Gong, Hui Chen, Huaijin Guan, Shaobo Su, Keqiang Chen, Ji Ming Wang


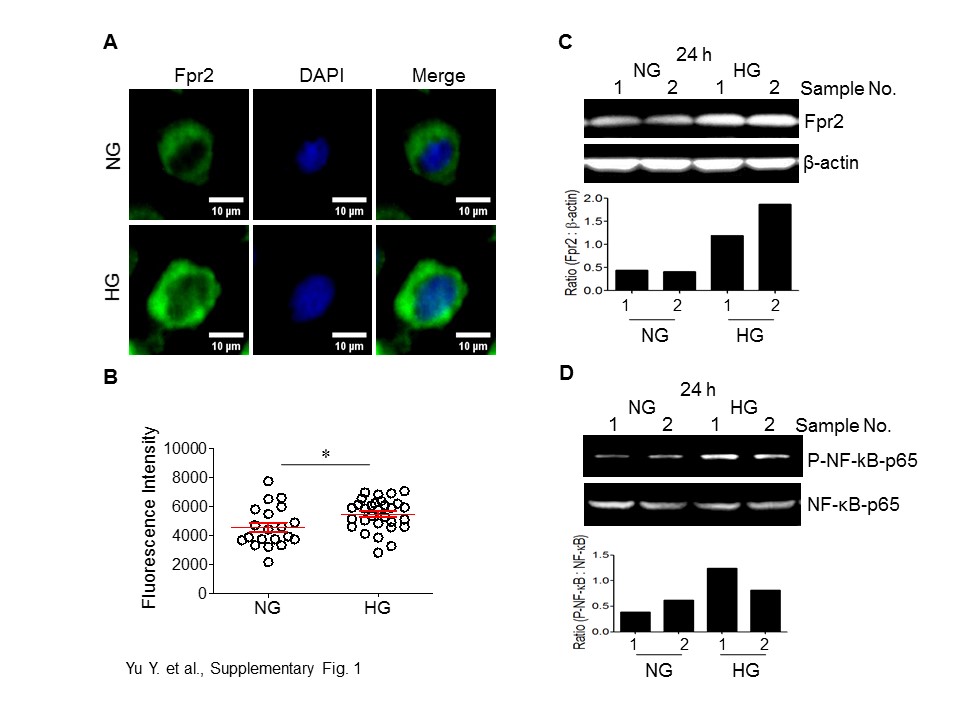


**Supplementary Fig. 1: The expression of Fpr2 by mouse MGCs**

MGCs seeded at 1.0×10^4^ cells/well on 8-well chamber slides for 24 h were exposed to normal glucose (5.5 mM, NG) or high glucose (25.0 mM, HG) for 24 h. **A.** Fpr2 fluorescence. MGCs were washed twice with PBS and fixed for 10 min with 4% paraformaldehyde in PBS, followed by Triton X-100 (0.1%) for 15 min. The cells were then incubated with 5% BSA in PBS for 1 h followed by addition of primary anti-mouse Fpr2 antibody (M-70, Santa Cruz Biotechnology). Secondary antibodies coupled to Alexa Fluor® 488 (Abcam, Cambridge, UK) were then added to the culture. After staining with DAPI to visualize nuclei, the cells were analyzed under a fluorescence microscope (Olympus IX 71). Green: Fpr2; Blue: Nuclei; Scale bar: 10 μM. **B.** Fluorescence intensity of Fpr2 on MGCs. Cell numbers: 20-30 for NG or HG treatment. *p < 0.05. **C.** Western blotting showing Fpr2 protein levels in MGCs treated with NG or HG for 24 h. **D.** Western blotting showing phosphorylation of NF-κB-p65 in MGCs cultured in NG or HG for 24 h.


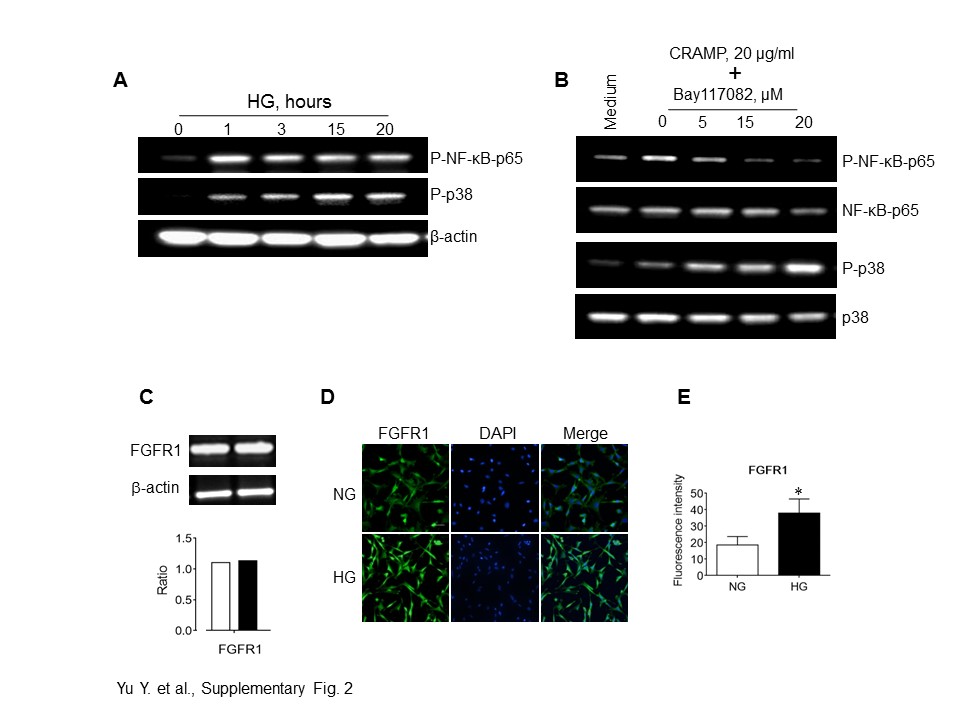


**Supplementary Fig. 2: NF-κB-p65 phosphorylation and FGFR1 expression in MGCs**

**A.** Western blotting showing phosphorylation of NF-κB-p65 in MGCs cultured in HG at the indicated time points. **B.** Western blotting showing the inhibition of CRAMP-induced NF-κB-p65 phosphorylation by BAY 11-7082 in MGCs in HG. **C-E.** FGFR1 expression in mouse MGCs. **C.** No difference in FGFR1 mRNA levels in MGCs cultured in HG or NG. **D.** Increased level of FGFR1 protein shown by fluorescence intensity in HG-tcultured MGCs. * indicates significantly increased (p < 0.05) FGFR1 fluorescence intensity in HG-treated MGCs compared with cells treated with NG.

**
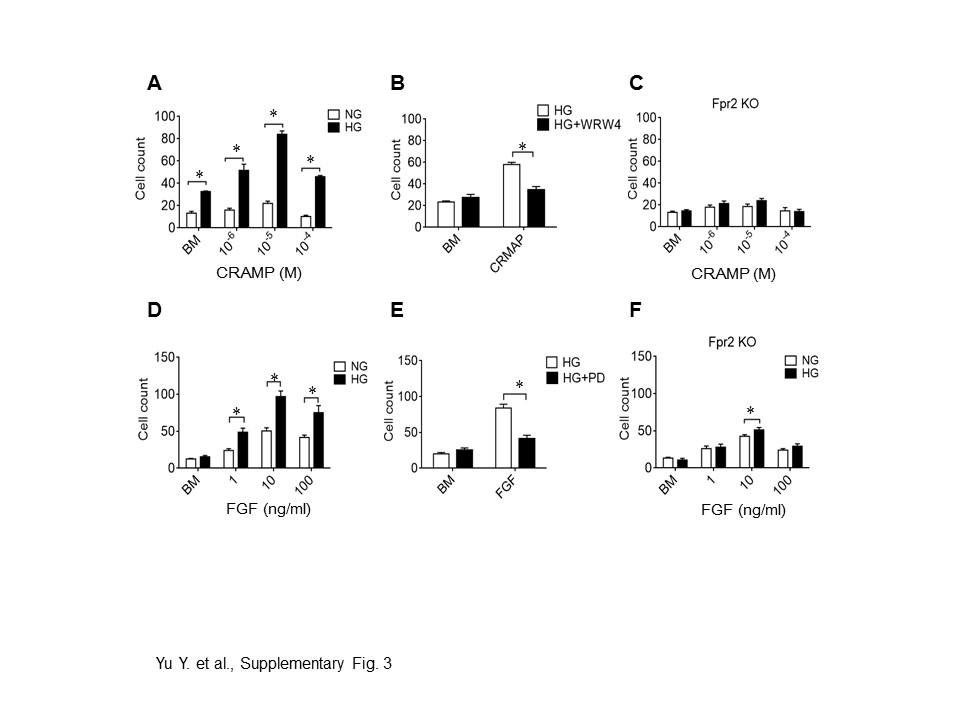
**

**Supplementary Fig. 3: Fpr2- and FGFR1- mediated chemotaxis of MGCs**

MGC chemotaxis was measured by 48 well chambers. The results are expressed by the mean + SD numbers of the cells migrated in response to chemoattractants. **A.** Migration of MGCs cultured with NG or HG in response to CRAMP. **B.** Inhibition of CRAMP- (10^-5^ M) induced chemotaxis of MGCs by the Fpr2 antagonist WRW4. **C.** Absence of chemotaxis of MGCs from Fpr2^-/-^ mice in response to CRAMP. **D.** Directional migration of MGCs in response to b-FGF (10 ng/ml). **E.** Inhibition of b-FGF-induced chemotaxis of MGCs cultured in HG by the FGFR antagonist PD 173074 (PD). **F.** Chemotaxis of MGCs from Fpr2^-/-^ mice in response to b-FGF. * p < 0.05.


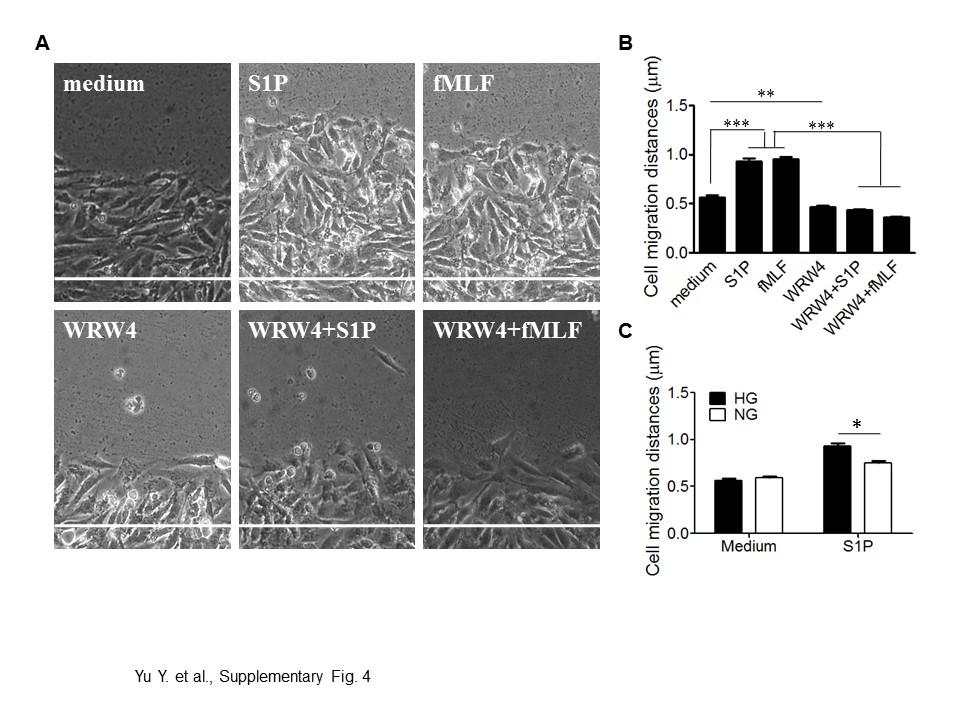


**Supplementary Fig. 4: S1P enhances the closure of MGC monolayer wound**

**A.** The effect of S1P (20 μg/ml) on MGC movement toward the centerline of the monolyer wound in HG. An Fpr2 ligand fMLF (10^-5^ M) as used as a control. **B.** Quantitation of the distances moved by MGCs shown in **A**. **C.** Quantitation of the distances moved by MGCs in the presence of S1P (20 μg/ml) shown in **A.** NG or HG. The effect of both S1P and fMLF was attenuated by the presence of the Fpr2 antagonist WRW4. *p < 0.05, ** p < 0.01, *** p < 0.001.


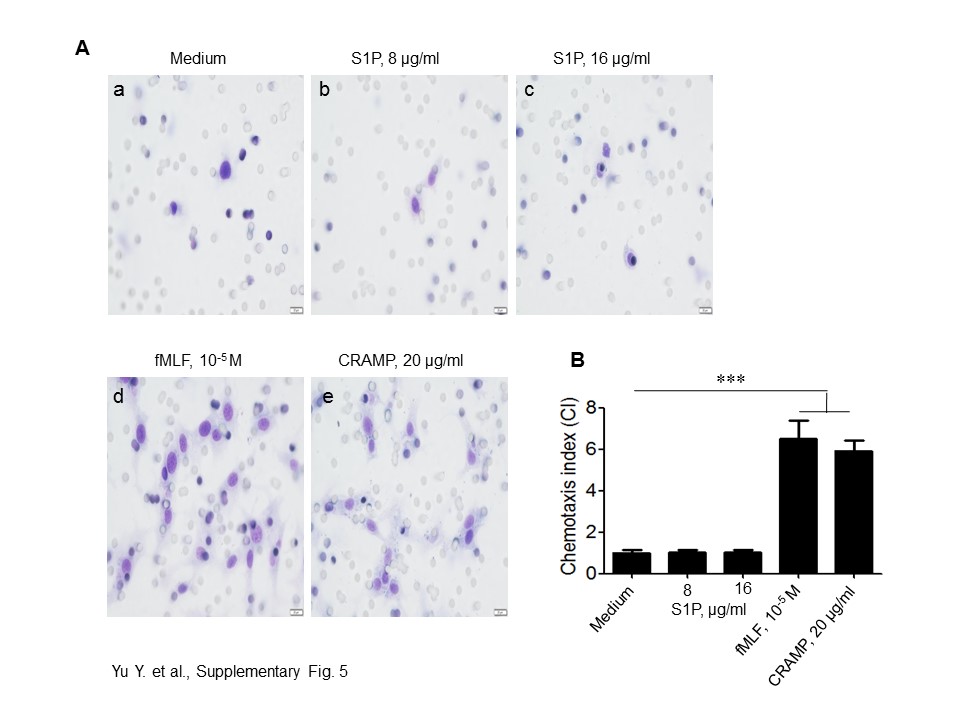


**Supplementary Fig. 5: S1P did not induce the directional migration (chemotaxis) of MGCs**

Chemotaxis assays for MGCs in response to S1P were performed with 48-well chemotaxis chambers in response to different concentrations of S1P. The Fpr2 ligands fMLF (10^-5^ M) and CRAMP (20 μg/ml) were used as controls. HG-treated MGCs in binding medium containing 1% BSA (50 μl, 1.8×10^6^/L) were placed in wells of the upper compartment**. A.** MGCs migrated across the chemotaxis membrane in response to stimulants. **B.** Chemotaxis indexes of MGCs in response to S1P, fMLF or CRAMP. *** p < 0.001.


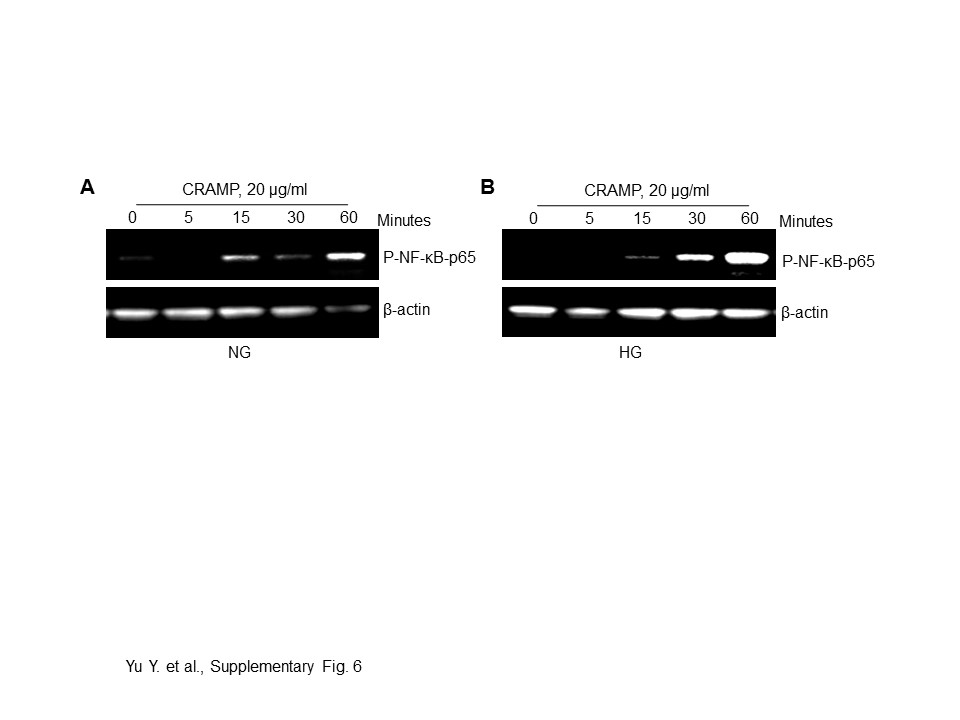


**Supplementary Fig. 6: HG increases the phosphorylation of NF-κB-p65 in MGCs in response to CRAMP**

**A.** Western blotting showing CRAMP-induced phosphorylation of NF-κB-p65 in MGCs cultured in NG. **B.** Western blotting showing CRAMP-induced phosphorylation of NF-κB-p65 MGCs cultured in HG.
